# Potential enhancement of intravenous nano-hydroxyapatite in high-intensity focused ultrasound ablation for treating hepatocellular carcinoma in a rabbit model

**DOI:** 10.3892/ol.2014.1900

**Published:** 2014-02-21

**Authors:** LIPING LIU, ZIWEN XIAO, YANBING XIAO, ZHIBIAO WANG, FAQI LI, MAOPING LI, XIAOQIONG PENG

**Affiliations:** 1 Department of Ultrasound, The First Affiliated Hospital of Chongqing Medical University, Chongqing 400016, P.R. China; 2 Department of Biomedical Engineering, Chongqing Medical University, Chongqing 400016, P.R. China

**Keywords:** nano-hydroxyapatite, hepatocellular carcinoma, high-intensity focused ultrasound, rabbit model

## Abstract

The aim of the present study was to evaluate the safety and efficiency of an intravenously delivered nano-hydroxyapatite (Nano-HA) solution into a rabbit model (*Oryctolagus cuniculus*) to determine the potential enhancement of high-intensity focused ultrasound (HIFU) for the ablation of hepatocellular carcinoma (HCC) in liver tissue. The present study clearly indicated that the intravenous delivery of large quantities of Nano-HA into the body of the rabbit model over relatively short periods of time may be absorbed by the hepatic reticuloendothelial system. Subsequent HIFU treatment for HCC, as well as intravenous Nano-HA, produced a rapid increase in temperature and an enlargement of the coagulated necrotic area during ablation in the *in vivo* and *ex vivo* environments. In addition, it was found that the therapeutic doses of Nano-HA produced mild and transient abnormalities in the normal renal function and hepatic enzymes during the first 24 h following administration. The results of the current study indicated that the combination of Nano-HA and HIFU may provide a safe and effective alternative to conventional surgical procedures.

## Introduction

Hepatocellular carcinoma (HCC), also termed malignant hepatoma, is the most common type of liver cancer and the sixth most common type of cancer worldwide (1). The majority of patients with HCC are diagnosed at an advanced stage and therefore, only 10–15% of patients are suitable for surgical resection (2). High-intensity focused ultrasound (HIFU), which was developed in the 1940s as a viable thermal tissue ablation approach, is a recently developed local ablation technique for the clinical management of solid tumors, particularly for unresectable HCC cases (3,4). Although the initial experience regarding its efficacy has been promising, its clinical application remains limited, particularly when treating large tumors of a significant depth. In order to improve the efficacy of the HIFU technique, intensive efforts have been made in recent years to identify an optimal exogenous enhancer.

Currently, nanoparticles, with well-defined inner and outer surfaces, may be easily functionalized for biological applications and have attracted detailed attention in biotechnological studies (5). The application of nanoparticles as disease-specific imaging contrast agents has been popular (6) thus, the possibility of developing a nanoparticle-based ultrasound contrast agent is increasing (7). Among nanoparticles with different material composition, inorganic nanoparticles that are composed of calcium phosphate have numerous advantages, including ease of synthesis, control of physicochemical properties, marked interactions with their payload and satisfactory biocompatibility (8). The smaller size of nano-hydroxyapatite (Nano-HA) compared with red blood cells contributes to its ability to freely transfuse in the blood cycle *in vivo*. In addition, as previously reported, Nano-HA may significantly inhibit the *ex vivo* and *in vivo* growth of the human hepatoma cell line, BEL-7402, by inducing tumor apoptosis and decreasing proliferation with no or marginal damage to human normal hepatic cells (9). Therefore, it is reasonable to hypothesize Nano-HA as an exogenous enhancer to HIFU treatment; Nano-HA may enhance the treatment efficiency of HIFU markedly by changing the acoustic environment of the tissue with the local accumulation of Nano-HA. Thus far, previous experimental studies have rarely reported on the enhancement of intravenous Nano-HA in cancer ablation using HIFU.

The present study was designed to investigate the safe use of an intravenously delivered Nano-HA solution into a rabbit model (*Oryctolagus cuniculus*) to determine its potential enhancement of HIFU for the ablation of HCC. Initially, the *ex vivo* stability of the Nano-HA solution was determined and the *in vivo* safety of the intravenous delivery of large quantities of Nano-HA was established. Secondly, the optimal Nano-HA concentration of intravenous Nano-HA in liver ablation by HIFU was estimated. Finally, *ex vivo* and *in vivo* therapeutic effects of HIFU treatment for HCC, using Nano-HA, were evaluated using VX2 carcinoma in rabbit livers as a model. The results indicated that the combination of Nano-HA and HIFU may be a safe and effective alternative to conventional surgical procedures.

## Materials and methods

### Ethical approval

The procedures using rabbits were conducted according to the relevant national and international guidelines. The animal protocol was approved by the Institutional Animal Care and Use Committee according to the guidelines of the Association for the Assessment and Accreditation of Laboratory Animal Care International.

### Animals and materials

The animal study was approved by the Chongqing Experimental Animal Committee (Chongqing, China). The New Zealand white rabbits of mixed gender (1.8±0.1 kg) were provided by the Experimental Animals Centre of Chongqing Medical University (Chongqing, China) and kept in a controlled environment with free access to food and water. The rabbits were sacrificed according to the appropriate ethical guidelines. The surgical procedures were performed under sterile conditions and the rabbits were placed under an intramuscular anesthesia, which comprised of 5 mg/kg xylazine, 50 mg/kg ketamine and 10 mg/kg acetylpromazine. The extent of anesthesia was monitored by observing the heart and respiratory rates, the eye reflex and response to a stimulus. The surgical site was shaved and cleaned using povidone-iodine solution (Betadine, Purdue Pharma L.P., Stamford, CT, USA) and 0.25% Marcaine without epinephrine was administered subcutaneously as a local anesthetic.

The needle-like Nano-HA composites (diameter, ~12.00–19.33 nm and length, ~42.6–183.2 nm) were synthesized using a hydrothermal method and provided by the Sichuan University Biomaterials Engineering Research Center (lot no. 3030002; Chengdu, China). Lecithin, sodium carboxymethyl cellulose, glycerine, acetic acid glacial, saline, calcium nitrate, ammonium phosphate and arginine (Sigma-Aldrich, St. Louis, MO, USA; Shanghai Chemistry Reagent Company, Shanghai, China) were the materials that were used.

### Instruments

The contact thermometer, TFD-04 (Fudan University, Shanghai, China), CZF ultrasonic therapeutic apparatus (output power, 1–5 W; Chongqing Haifu Technology Co. Ltd., Chongqing, China) and Hitachi H-600 transmission electron microscope (S-4000, Hitachi, Ltd., Tokyo, Japan) were provided by the Institute of Ultrasonic Engineering in Medicine located at the Chongqing Medical University (Chongqing, China). The mixtures to suspend Nano-HA within the various solvents were sonicated using an XL2020 sonicator microtip (Heat Systems, Farmingdale, NY, USA), and the biochemical indicators of multiple organ function were analyzed using a Beckman CX7 analyzer and Beckman reagents (Beckman Coulter, High Wycombe, UK).

### Acute toxic detection of intravenous Nano-HA in a rabbit model

During the initial study on acute toxicity, intravenous Nano-HA was administered in the marginal vein of the ear. A total of 60 rabbits were randomly divided into group A (n=50) for investigating the lethal dose and group B (n=10) for evaluating the influences of the therapeutic dose of Nano-HA on the blood biochemistry parameters. Nano-HA was diluted using 0.9% saline to a concentration of 20 g/l. Group A received a rapid infusion of 120–300 mg/kg Nano-HA with 1 ml saline (0.9%) sequentially into the rabbit auricular vein, followed by two-weeks of follow-up treatment. Group B received a rapid infusion of 50 mg/kg Nano-HA and 1 ml saline (0.9%) sequentially into the auricular vein. Blood samples were subsequently obtained to detect liver and renal function, lactate dehydrogenase (LDH), creatine kinase (CK), magnesium, calcium and phosphorus at 15 min, 30 min, 1 h, 2 h, one day, three days, one week and two weeks following the Nano-HA intravenous infusion. The rabbit hearts, lungs, kidneys and livers were harvested to evaluate tissue pathological injuries.

### Influence of intravenous Nano-HA on in vivo liver ablation using HIFU in a rabbit model

The 80 rabbits were randomly divided depending on different Nano-HA doses and interval times following the Nano-HA intravenous injection. The parameters that were applied for *in vivo* HIFU tissue ablation were as follows: Generator power, ≤5 W; generator frequency, 6.0 MHz; focal length, ≤8 mm; and pulse duration, ≤10 secs. The Nano-HA was diluted using 0.9 % saline to a concentration of 20 g/l. At 24 h prior to the surgical procedures the rabbits in the Nano-HA groups received a rapid infusion of different doses of Nano-HA and 1 ml saline (0.9%), sequentially. The rabbits in the control group received a rapid infusion of 2 ml/kg saline (0.9%) in the auricular vein. The rabbits underwent a median laparotomy to expose the liver through an 8-cm incision upon the liver site. Following the *in vivo* HIFU intervention, the incision was closed and labeled with a 6:0 Prolene suture (Ethicon, Somerville, NJ, USA) under an intramuscular anesthesia of 5 mg/kg xylazine, 50 mg/kg ketamine and 10 mg/kg acetylpromazine. The rabbits were promptly restored by intramuscular injection of 0.1 ml/kg Suxingling. All of the experimental rabbits were subjected to an additional median laparotomy to expose the liver 24 h following the surgical procedures. The liver tissues were sampled and fixed with 5% glutaric dialdehyde for scanning electron microscopy and 10% paraformaldehyde for light microscopy (VH-X, Keyence Corporation, Osaka, Japan). The rabbits were sacrificed by a rapid infusion of 10 ml air into the auricular vein. Subsequently, the whole livers were harvested and analyzed. The volumes of coagulation necrotic loci under HIFU were calculated using the following formula: Volume (mm^3^) = πabh/6, where a indicates the major axis, b the minor axis and h the depth perpendicular to the plane of the sound channel. The energy efficiency factor (EEF) was evaluated and calculated using the following formula: EEF = ηPT/V, where η indicates the energy efficiency coefficient, P the generator power, T the pulse duration time and V the volume of coagulation necrotic loci.

### In vivo influence of intravenous Nano-HA on ablated HCC using HIFU in a rabbit model

The rabbit VX2 carcinoma cell line (Funabashi, Kyoto, Japan) proliferated rapidly in the tissue culture flasks containing Dulbecco’s modified Eagle medium/nutrient mixture and F-12 medium supplemented with 10% fetal bovine serum (FBS). An intramuscular injection of the VX2 cell line was administered in the thigh muscle (vastus lateralis) of a New Zealand white male rabbit to establish a tumor donor rabbit. The tumor was harvested after four weeks and minced into 1.0-mm^3^ cubes of tissue and stored in FBS at −70°C with 10% dimethyl sulfoxide until implantation. On the day of tumor implantation, the 1-mm^3^ slice grafts were thawed out and washed three times in Hanks’ buffered salt solution. During a minilaparotomy procedure, the tissue grafts were implanted into the left liver lobe of 30 recipient rabbits (weight, 2.50–3.30 kg) using non-invasive ultrasound imaging to demonstrate the tumor growth. In total, 35 tumor recipient rabbits underwent implantation of VX2 tumors and each received an equal tumor load.

**Figure 1:**
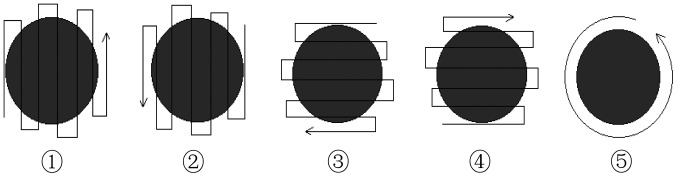
Exposure methods of focal ultrasound to treat liver cancer *in vivo.*

The tumor recipient rabbits were randomly divided into the following groups: Group A (n=10), without any therapeutic administration; group B (n=10), *in vivo* HIFU irradiation 18 days following VX2 liver tumor implantation; and group C (n=10), 50 mg/kg Nano-HA intravenous injection 18 days following VX2 liver tumor implantation, and *in vivo* HIFU irradiation 24 h later. All of the irradiated rabbits underwent a median laparotomy to expose the liver directly to the HIFU. The parameters of the HIFU that were applied for the *in vivo* liver tumor ablation were as follows: Generator power, ≤5 W; generator frequency, ≤6.0 MHz; focal length, ≤8 mm; and pulse duration, ≤300 sec. HIFU scanning was performed according to a sequence of five steps, with 60 sec per step (Fig. 1).

### Ex vivo influence of intravenous Nano-HA on ablated HCC using HIFU in a rabbit model

As described above, 30 tumor recipient rabbits (weight, 2.50–3.00 kg) underwent implantation of VX2 tumors. All of the rabbits received an equal tumor load. The tumor recipient rabbits were randomly divided into the following groups: Group A (n=10), without any therapeutic administration; group B (n=10), *ex vivo* HIFU irradiation 18 days following the implantation of VX2 liver tumors; and group C (n=10), 50 mg/kg Nano-HA intravenous injection 18 days following the implantation of VX2 liver tumors, and *ex vivo* HIFU irradiation 24 h later. The parameters of the HIFU treatment that were applied for *ex vivo* tumor ablation were as follows: Generator power, ≤200 W; generator frequency, ≤0.7 MHz; focal length, ≤135 mm; and pulse duration, ≤100 sec in the model of rectilinear 3D ultrasound scanning (rectilinear length, 10 mm and rate, 3 mm/sec. The entire scanning range completely covered the tumor loci and 5–10 mm beyond the tumor border.

### Histological examination

Following the mortality or sacrifice of the rabbits, the tissues were harvested between 2 h and one week subsequently. Following overnight fixation, the tissues were trimmed, sectioned at the widest margin, embedded in paraffin and sectioned in 5-μm increments. The sections were performed perpendicularly to the anterior-posterior axes. A total of three sections were placed on a slide and stained with hematoxylin and eosin, as well as periodic acid-Schiff reagent. Of the three sections on any one slide, the sections with the widest tissue parenchyma were used for the assessment. Fine structures within the liver were observed using electron microscopy.

### Statistical analysis

Data were analyzed using SPSS software 16.0 (SPSS, Inc., Chicago, IL, USA). The two-tailed unpaired Student’s t-test was used for comparison between the groups and subgroups. P<0.05 was considered to indicate a statistically significant difference.

## Results

### The contents and stability of certain Nano-HA compounds

To prevent a microvessel embolism, which may be induced from intravenous injection of solid-phase Nano-HA, Nano-HA suspensions containing certain organic and inorganic solvent compounds (5–6.5 pH) were oscillated for 10 min and assessed for stability. As shown in Table I, Nano-HA diluted with 0.9% saline to the concentration of 1 g/l was applied as this was reliable and exhibited optimal stability with the longest time of *ex vivo* precipitation conformation.

### Acute toxicity and serum biochemical influence of intravenous Nano-HA in a rabbit model

The rabbits were randomly divided into group A (n=50) for exploring lethal dose and group B (n=10) for evaluating influences of blood biochemistry parameters. Following the intravenous Nano-HA injection and during the follow-up period, clinical evidence of mortality parameters were noted, including aggressive listlessness, unstable gait, shortness of breath, screaming, convulsions, general weakness, cyanosis and loud breathing.

At 24 h following the Nano-HA injection, all of the rabbits in group A that survived, did so without evident side-effects and were administered additional injections during the two-week follow-up. As shown in Table II, the LD_50_ was 200 mg/kg.

In group B, the biochemical indicators of liver function demonstrated that the levels of alanine aminotransferase (ALT), aspartate aminotransferase (AST) and alkaline phosphatase (ALP) markedly increased following intravenous Nano-HA injection (0 h) and gradually returned to the normal range three days later. ALT and AST peaked at 2 h, while ALP peaked at 24 h as shown in Table III. The glutamyl transpeptidase level was markedly below the normal lower limits at 1–2 h and returned to the normal range at 24 h. No significantly abnormal changes in the total protein and albumin levels were observed during Nano-HA administration or the two-week follow-up.

Table IV shows the biochemical indicators of renal function and serum electrolyte alterations, demonstrating that the blood urea nitrogen level gradually increased following the Nano-HA injection, peaked at 24 h and rapidly decreased to the normal range three days later. The levels of creatine, LDH and CK markedly increased following the intravenous Nano-HA injection, peaked at 1–2 h and gradually returned to the normal range three days later. The serum magnesium, calcium and phosphorus concentrations were almost maintained at the normal range during Nano-HA administration and the two-week follow-up.

**Figure 2:**
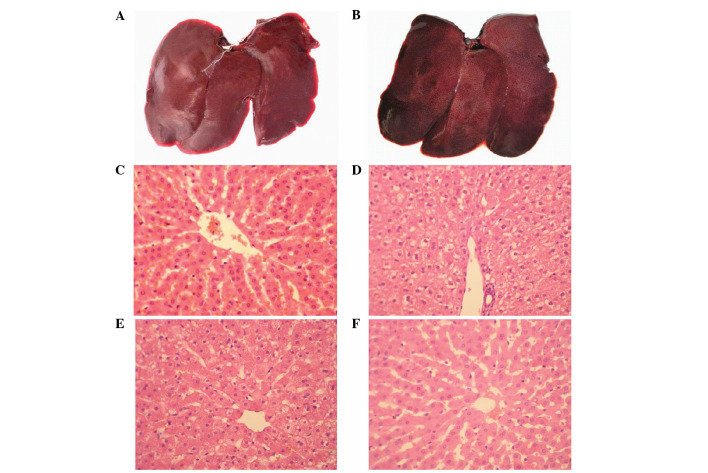
Liver tissue gross anatomy and structures were not clearly altered following Nano-HA administration in a rabbit model observed by light microscopy. (A) Normal liver tissue. (B) Liver of rabbits 24 h following the Nano-HA intravenous injection. (C) Normal liver tissue. Lightly stained liver cells and a loose cytoplasm were identified (D) 24 h and (E) one week following the Nano-HA intravenous injection. (F) Liver tissue and cells were normal two weeks following Nano-HA intravenous injection (magnification, ×400). Nano-HA, nano-hydroxyapatite.

**Figure 3:**
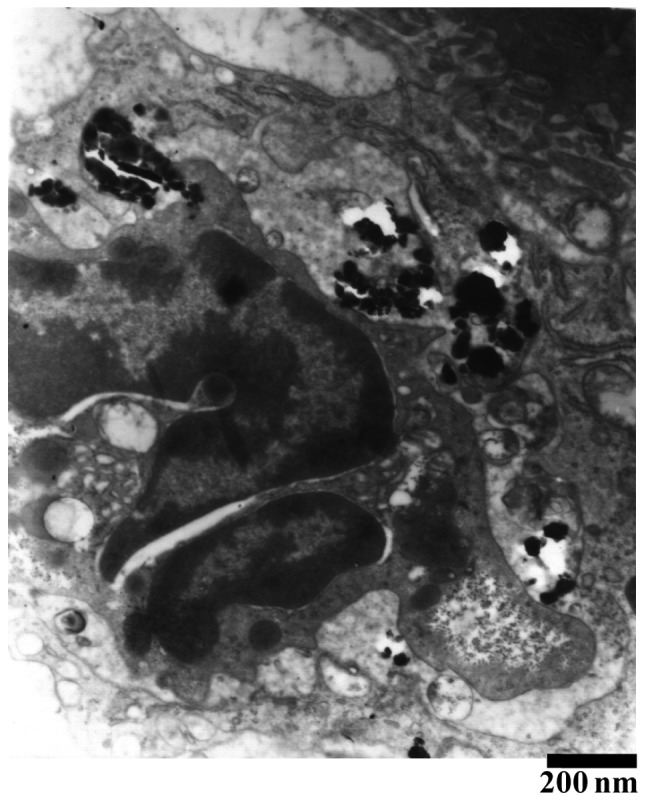
A large quantity of refracting crystal granules were detected by electron microscopy within the Kupffer cells ≥24 h following the nano-hydroxyapatite intravenous injection.

Additionally, as shown in Fig. 2 for group B, 2 h following the intravenous Nano-HA administration, no marked changes to the gross tissue anatomy were identified in the liver. One day later, small irregular nodules composed of neogenetic hepatic lobules emerged on the liver surfaces, while hepatic cells were slightly stained with a vesicular, pale and watery cytoplasm. The hepatic vesicular contents evidently decreased in the following two days and returned to normal two weeks following the intravenous Nano-HA administration. In addition, as shown in Fig. 3, a large quantity of refracting crystal granules were detected within the Kupffer cells ≥24 h following the Nano-HA intravenous injection.

### Enhancement of intravenous Nano-HA during in vivo HIFU ablation in a rabbit model

Initially, 40 rabbits were randomly divided into 50, 100, and 150 mg/kg Nano-HA treatment and control saline groups. The temperature of the ultrasound irradiated sites significantly increased in a Nano-HA dose-dependent manner, as shown in Table V. The liver sites that were subjected to HIFU irradiation showed gray coagulation necrosis. Compared with the saline control, the coagulation necrotic volumes induced by HIFU were significantly enlarged in a Nano-HA dose-dependent manner (P<0.05), while EEF was significantly decreased in a Nano-HA dose-dependent manner (P<0.05). As shown in Table VI, an additional 40 rabbits were randomly divided into groups dependent on the level of Nano-HA dosage at 0 min, 30 min, 2 h, 24 h, 48 h, 72 h, one week and two weeks following the 50 mg/kg Nano-HA intravenous injection. Accompanying the corresponding reduction of EEF (P<0.05), the irradiated temperature and coagulation necrotic volumes under HIFU significantly increased between 2 h and one week following Nano-HA intravenous injection (P<0.05) and peaked at 48 h following the Nano-HA injection (P<0.05).

### In vivo enhancement of intravenous Nano-HA in ablated HCC using HIFU in a rabbit model

Among the tumor recipient rabbits, group A (n=10) received no therapeutic administration, group B (n=10) underwent *in vivo* HIFU irradiation and group C (n=10) underwent a combination of Nano-HA and the *in vivo* HIFU irradiation.

**Figure 4:**
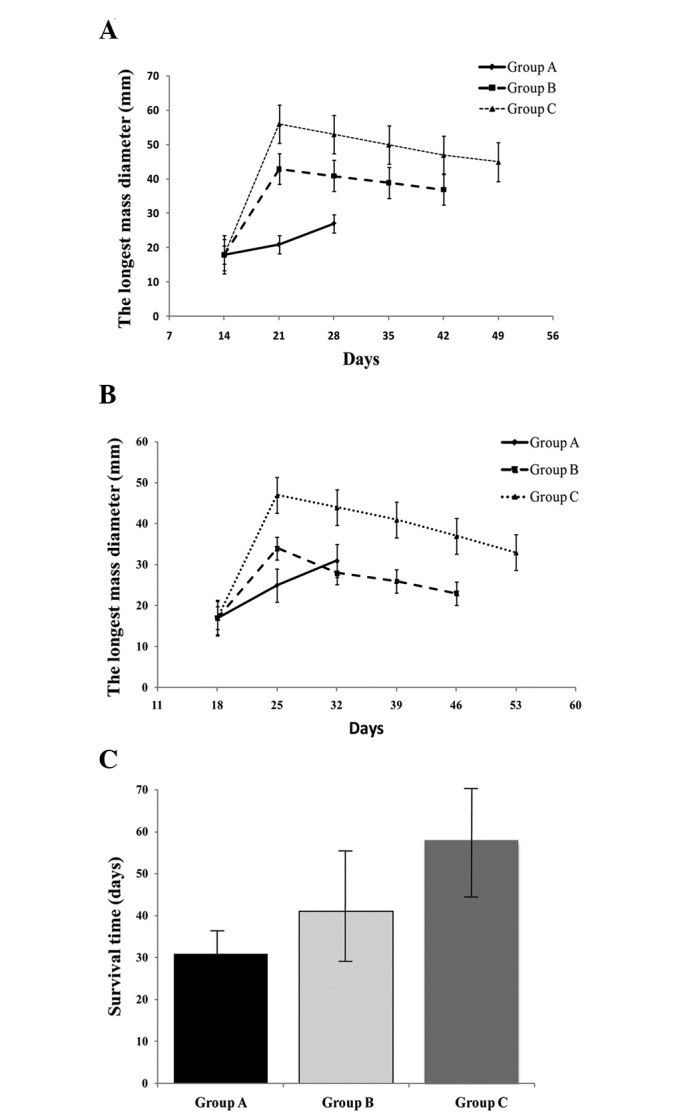
Different factors of the rabbits that were implanted with VX2 cancer in the liver, which was influenced by Nano-HA and Nano-HA *ex vivo* HIFU. Group A received no therapeutic administration, group B underwent *in vivo* HIFU irradiation and group C underwent a combination of Nano-HA and *in vivo* HIFU irradiation, (n=10). The longest diameters of the tumor and irradiated loci in the ultrasound images were observed in group C, followed by groups B and A at the same time-interval following the treatment intervention. (A) Ultrasound images of the tumor and irradiated loci of the rabbits that were implanted with VX2 cancer in the liver, which was influenced by Nano-HA *in vivo* HIFU. (B) Diameter of the tumor and irradiated loci of the rabbits that were implanted with VX2 cancer in the liver, which was influenced by Nano-HA *ex vivo* HIFU. (C) Survival time of the rabbits that were implanted with VX2 cancer in the liver, which was influenced by Nano-HA and/or *ex vivo* HIFU. Nano-HA, nano-hydroxyapatite; HIFU, high-intensity focused ultrasound.

Tumor growth was observed in all of the30 rabbits, showing visible tumorous nodules on the second laparotomy. The rabbits presented no outward signs of sickness or abdominal lymph node metastases prior to sacrifice, while one rabbit in group A and one rabbit in group C showed abdominal wall metastases. As shown in Fig. 4A, the tumor volumes rapidly increased to comparable sizes between each group. The longest diameters of the tumor and irradiated loci in the ultrasound images were significantly longer when comparing group C with group B at the same time interval following HIFU irradiation (P<0.05). The longest survival time of 63.50±21.99 days was detected in group C, which was significantly longer than 47.50±12.79 days, which was detected in group B and 30.00±7.60 days, which was detected in group A (P<0.05). Furthermore, three rabbits in group C were kept alive for three months until they were sacrificed.

### Ex vivo enhancement of intravenous Nano-HA in ablated HCC using HIFU in a rabbit model

Among the tumor recipient rabbits, group A (n=10) received no therapeutic administration, group B (n=10) underwent *ex vivo* HIFU irradiation and group C (n=10) underwent a combination of Nano-HA injection and *ex vivo* HIFU irradiation.

Tumor growth was found in all 30 rabbits showing visible tumorous nodules following the second laparotomy. In group A, the longest tumor axis markedly increased beyond 18, 25 and 32 days following implantation of VX2 tumors (P<0.05) and nine rabbits presented with abdominal metastasis at day 25. Notably, two weeks following HIFU irradiation, the longest axis of tumors and irradiated loci began to decrease in groups B and C, as shown in Fig. 4B. Abdominal or hepatic metastasis occurred in eight rabbits in group B between 39 and 46 days following implantation of VX2 tumors, while in group C, abdominal metastasis occurred in only three rabbits after day 39, two rabbits after day 46 and three rabbits after day 60. In addition, the two remaining rabbits survived without metastasis for >three months. As shown in Fig. 4C, the overall survival rate was significantly higher when comparing groups B or C to group A (P<0.05) and group C to group B (P<0.05).

## Discussion

Extracorporeal ultrasound-guided HIFU was initially introduced in the late 1990s as a clinical treatment for solid tumors, including pancreas, liver, prostate, breast, uterine fibroid and soft-tissue sarcomas (10). The principal mechanisms of HIFU are coagulative thermal necrosis, which results from the absorption of ultrasound energy during transmission in the tissue and the induced cavitation damage (11). Therefore, HIFU treatment, based on the application of heat and cavitation, may be performed as a minimally invasive option with low morbidity, no invasion, no ionization, fewer complications following treatment and simple post-treatment management. Recently, HIFU has been approved in China and is used as an alternative to surgery for solid tumors (12). However, the preliminary trials for liver-cancer treatments, indicated that the predominant limitations of HIFU were its incomplete treatment (or treatment failures), particularly when facing large tumor masses and/or tumor masses with a significant depth (13). Therefore, the increase of HIFU effectiveness, which may improve the therapeutic efficacy with few side-effects, has been a predominant area of focus. Notably, with a suitable contrast agent, the contrast in tumor areas may be greatly enhanced and monitored by using HIFU medical imaging facilities.

With the development of shell materials and preparation technologies, microbubble contrast nanoparticles, sized between 100 and 1 nm, as a dynamic indicator of tissue perfusion (14), have been enormously popular in molecular imaging (15) and drug delivery, as well as enlarging the ablated volume within the target liver tumors using HIFU (16). The nanoparticle properties of small-size and preferential accumulation at tumor sites, due to the absence of an effective lymphatic drainage system in tumorous masses, identified them to be useful in oncology, particularly in imaging. This indicated that microbubble contrast nanoparticles may be administered safely as an optimal enhancer for medical ultrasound interventions for the treatment of solid malignancies. Conversely, previous clinical trials have confirmed that osteosarcoma is the optimal neoplasm for receiving HIFU treatment (17) and these results, to some extent, indicated that the bone-specific content of hydroxyapatite may be sensitive to the therapeutic efficacy of HIFU (18). In addition, Nano-HA has already been used as a satisfactory biological material for filling bone defects owing to its good biological activity, biocompatibility and conjunctive ability with bone tissues (19). Nano-HA composites do not release HA *in vivo* and may be excreted from the urine via glomerular filtration. For these abovementioned reasons, it was hypothesized that Nano-HA composites may be a safe, effective and feasible alternative to improve the limited HIFU ablation for HCCs.

The present study demonstrated for the first time that intravenous delivery of abundant Nano-HA may be assembled by the hepatic reticuloendothelial system within a short time period, subsequently leading to a rapid increase of ultrasound-induced overheating, which consequently enlarges the coagulation necrotic area for HCC when ablated by HIFU in rabbits *in vivo* (Fig. 6A) and *ex vivo* (Fig. 6B). In addition, the therapeutic doses of injected Nano-HA were markedly lower than the lethal doses (Table II), only presenting transient and mild influences on the hepatic renal function and electrolyte balance during the initial 24 h following the Nano-HA injection (Table II). No Nano-HA absorption within the hepatic cells, and only reversible and mild morphological alterations were observed following the intravenous delivery of abundant Nano-HA particles (Fig. 2). All of the abovementioned results indicate that it is reliable and safe to combine HIFU with intravenous Nano-HA. Furthermore, Nano-HA application decreased EEF when the therapeutic volumes were increased by the HIFU treatment, which was dependent on the Nano-HA dose (Table VI), indicating the satisfactory efficacy of combining HIFU with intravenous Nano-HAs.

The mechanisms of the enhancement of intravenous Nano-HA in ablated HCC by HIFU has been elucidated in the present study, however, the results may be interpreted as follows (although the Nano-HA particles were absorbed by hepatic kupffer cells rather than hepatic VX2 cells): i) Globoids possessing millions of Nano-HA particles assembled within the sinus hepaticus were completely scattered around each hepatic cell, which altered the hepatic characteristic impedance and back scatter coefficient of the sound waves; ii) the absorbed Nano-HA particles altered the hepatic structure and function, and influenced the sonic flow and the attenuation coefficient when the sound waves passed through the hepatic tissue; and iii) the hepatic Nano-HA particles that possessed innate high non-linear parameters may have increased exponentially to form large levels of higher harmonics with which more sonic power may have effectively been transformed into thermal power to increase the therapeutic damage to the tumor. Thereby, additional studies are required to confirm the abovementioned hypotheses and the manner in which Nano-HA is assembled by the hepatic reticuloendothelial system.

Of note, the entire parameter set that was applied in the present study was based on a previous rabbit model. The safety and efficacy of the combination of intravenous Nano-HA and HIFU in ablated HCC requires further investigation and should be restricted to carefully selected human cases in the future.

The goal of the current study was to evaluate intravenous Nano-HA to enhance HCC ablation by HIFU in a rabbit model. The results confirmed the hypothesis that a combined application of Nano-HA and HIFU may be a more effective and alternative tool for HCC local ablation in a safe and feasible manner compared with the current surgical procedures. These results may enable further investigations to identify the underlying mechanisms on enhanced tumor ablation by the combined application of Nano-HA and HIFU, as well as extend the applicable range of Nano-HA in the treatment of other solid tumor types.

## Figures and Tables

**Table I:** Contents of certain nano-HA compounds and their stability.

Nano-HA concentration (g/l)	Solvent concentration	Other content	Appearance	Stability (time of precipitation-conformation)
1	0.9% Saline	None	Bright and transparent liquid	>1 month
20	0.9% Saline	None	Milk-white suspended liquid	1 h
20	0.3% CMC-Na	Distilled water	Milk-white suspended liquid	12 h
20	0.3% lecithin + 1% glycerol	Distilled water	Milk-white suspended liquid	12 h
20	0.3% lecithin + 0.3% CMC-Na	Distilled water	Milk-white suspended liquid	12 h

Nano-HA, nano-hydroxyapatite; CMC-Na, sodium carboxyl methyl cellulose.

**Table II:** Mortality rates of rabbits with different toxicant dosage concentrations of Nano-HA.

Dosage of Nano-HA (mg/kg)	n	Mortality in 24 h (n)	Mortality (%)	Survival time	

				Mean (min)	Range
300	3	3	100	2.1±0.3	1–3 min
280	3	3	100	2.5±0.5	1–3 min
260	5	5	100	5.2±1.3	2–8 min
240	6	4	66.7	282±65.3	3 min–20 h
220	6	3	50	402.7±51.6	3 min–20 h
200	6	3	50	401.7±52.2	5 min–18 h
180	8	3	37.5	6.7±3.6	3–12 min
160	6	0	0		
140	4	0	0		
120	3	0	0		

Nano-HA, nano-hydroxyapatite.

**Table III:** Influence of Nano-HA on liver function in the rabbit model.

Group	TP (g/l)	ALB (g/l)	ALT (μl)	AST (μl)	ALP (μl)	GGT (μl)
Control	59.67±11.34	32.00±2.37	63.67±13.16	61.17±26.03	119.00±30.98	5.17±1.63
15 min	70.05±5.38	40.02±1.35	37.00±11.38	21.25±2.69	133.05±25.66	4.88±0.68
30 min	61.06±12.46	38.11±1.56	55.01±13.64	29.08±5.48	140.08±33.86	5.95±1.23
1 h	67.24±5.20	37.06±2.21	138.15±8.75	670.00±35.46	140.03±35.28	5.02±0.15
2 h	66.37±7.40	36.34±9.50	184.00±18.76	1027.00±205.87	147.00±29.74	4.50±0.13
24 h	54.67±0.57	33.67±1.53	120.67±83.05	163.00±20.25	163.33±65.03	5.00±3.00
3 days	62.00±6.08	35.00±2.65	64.00±16.37	41.00±11.53	118.67±18.72	5.67±3.51
1 week	53.50±2.65	30.50±2.08	55.75±15.82	27.75±3.30	88.25±22.38	5.00±1.41
2 weeks	57.25±15.76	30.25±6.40	77.75±14.98	69.50±30.03	80.25±38.80	4.50±2.19

Nano-HA, nano-hydroxyapatite; TP, total protein; ALB, albumin; ALT, alanine aminotransferase; AST, aspartate aminotransferase; ALP, alkaline phosphatase; GGT, glutamyl transpeptidase;

**Table IV:** Influence of Nano-HA on renal function and serum electrolytes in the rabbit model.

Group	BUN (mmol/l)	CRE (μmol/l)	LDH (μl)	CK (μl)	Mg (mmol/l)	Ca (mmol/l)	P (mmol/l)
Control	9.07±2.89	53.17±21.29	267.33±235.6	2291.03±1125.03	0.63±0.38	3.07±0.21	3.01±0.25
15 min	8.05±1.25	101.00±23.55	217.05±56.87	1558.12±96.87	0.51±0.21	3.05±0.36	2.53±0.55
30 min	8.70±1.13	112.02±12.48	189.00±32.65	1549.02±369.47	1.05±0.46	3.04±0.28	2.21±0.16
1 h	7.10±2.31	112.05±15.69	1378.00±102.89	2526.05±561.30	1.43±0.16	2.75±0.19	3.95±0.12
2 h	8.80±1.56	113.00±9.86	1474.21±68.77	3033.00±1234.26	1.25±0.32	2.87±0.31	4.34±0.58
24 h	12.33±3.68	107.00±40.60	421.00±94.60	2907.00±251.05	0.76±0.20	2.58±0.23	2.66±0.40
3 days	9.07±0.93	72.33±10.97	198.05±23.64	1582.67±427.68	0.73±0.22	2.84±0.14	2.76±0.13
1 week	6.40±3.80	82.25±12.50	199.50±178.92	1300.25±724.05	0.58±0.12	2.92±0.29	2.05±0.22
2 weeks	6.50±1.49	62.5±7.94	219.25±132.03	1851.26±1292.04	0.81±0.36	2.73±0.15	1.83±0.29

Nano-HA, nano-hydroxyapatite; BUN, blood urea nitrogen; CRE, creatine; LDH, lactate dehydrogenase; CK, creatine kinase; Mg, magnesium; Ca, calcium; P, phosphorus.

**Table V:** Enhancement of the *in vivo* HIFU ablation in a Nano-HA dose-dependent manner.

Drug	Dosage	n	Temperature increase (°C)	Volume (mm^3^)	EEF (J/mm^3^)
Saline	2 ml/kg	10	35.83±2.85	95.58±21.49	0.386±0.098
Nano-HA	50 mg/kg	10	40.97±2.35^a^	186.75±42.73^a^	0.194±0.034^a^
Nano-HA	100 mg/kg	10	41.68±2.48^a^	230.16±54.27^a^	0.161±0.036^a^
Nano-HA	150 mg/kg	10	43.06±3.72^a^	280.11±49.10^a^	0.128±0.019^a^

a P<0.05 compared with the saline control HIFU, high-intensity focused ultrasound; Nano-HA, nano-hydroxyapatite; EEF, energy efficiency factor.

**Table VI:** Influence of the *in vivo* HIFU ablation volume using 50-mg/kg Nano-HA at different time intervals.

Time following Nano-HA injection	n	Increased temperature (°C)	Volume (mm^3^)	EEF (J/mm^3^)
Control	5	35.69±2.82	95.58±21.49	0.39±0.09
30 min	5	36.58±2.39	111.53±27.13	0.324±0.047
2 h	5	38.95±3.65	137.24±40.29	0.265±0.067
24 h	5	40.97±2.27	186.75±42.73	0.19±0.03
48 h	5	41.40±2.86	201.39±51.26	0.182±0.035
72 h	5	39.67±1.88	180.68±24.47	0.197±0.025
1 week	5	38.93±2.28	165.99±20.92	0.215±0.027
2 weeks	5	37.82±2.16	105.52±14.84	0.338±0.047

Nano-HA, nano-hydroxyapatite; EEF, energy efficiency factor; HIFU, high-intensity focused ultrasound.
